# Rural-Urban Comparison of Health Risk Behaviors and Health Outcomes Among Chinese Older Adults: A Latent Class Analysis

**DOI:** 10.1007/s10823-026-09565-0

**Published:** 2026-03-18

**Authors:** Cai Xu, Yen-Han Lee, Mack Shelley, Peiyi Lu

**Affiliations:** 1 Department of Social Medicine, Heritage College of Osteopathic Medicine, Ohio University, Athens, OH 45701 USA; 2 Department of Health Sciences, College of Health Professions and Sciences, The University of Central Florida, Orlando, FL USA; 3 Department of Political Science, Department of Statistics, The Iowa State University, Ames, IA USA; 4 Department of Social Work and Social Administration, The University of Hong Kong, Hong Kong, SAR China

**Keywords:** Health-risk behavior, Chinese older adults, Health outcome, Latent class analysis

## Abstract

This study investigated health risk behavior patterns among older adults in rural and urban China and their association with health outcomes, addressing the limited evidence on rural–urban differences in health behaviors among Chinese older adults. We used cross-sectional data from the 2018 Chinese Longitudinal Healthy Longevity Survey. Latent class analysis identified latent classes of health behaviors (e.g., smoking, drinking, unhealthy diet), and regression models examined their associations with health outcomes, including indicators of physical health, perceived health, functional health, and mental health. Among 8,651 participants aged 65 and above (mean age = 82.9 years, SD = 11.2), distinct patterns of risky health behaviors were identified in both rural (*n* = 3,579) and urban (*n* = 5,072) samples, with combined unhealthy diet and substance use being most prevalent. Age and gender significantly predicted class membership. Rural participants engaging in smoking and drinking were more likely to experience chronic diseases, obesity, and functional limitations, while urban participants who consumed alcohol had greater risks of depression and anxiety. Distinct urban–rural differences in health behavior patterns were observed among older adults, and these patterns were differentially associated with health outcomes. These findings highlight the need for tailored interventions targeting different risk behaviors in rural and urban older adults in China, which may help reduce health inequalities and improve overall health.

## Introduction

Health behaviors encompass health-related practices that directly impact the health outcomes of individuals or communities (Y. B. o. Zhang et al., 2021). Healthy behaviors, such as regular exercise, balanced diets, and avoiding smoking, can lower the risk of chronic diseases like cardiovascular conditions and diabetes, while also improving mental and self-rated health (Conry et al., 2011). In contrast, unhealthy behaviors, including smoking, excessive alcohol use, and insufficient sleep, increase morbidity and mortality risks among adults (Cappuccio et al., 2010).

China’s rapidly aging population underscores the urgency of promoting healthy behaviors. By the end of 2022, 209.78 million people in China were aged 65 or above, accounting for 14.9% of the total population (National Bureau of Statistics of China, 2022). The aging trend is projected to increase the burden of chronic non-communicable diseases by at least 40% by 2030 (Wang et al., 2024). Findings from the 2018 Health-related Quality of Life Survey of Chinese adults aged 60 and above revealed a high prevalence of health risk behaviors, including physical inactivity (62.5%), unhealthy dietary habits (45.7%), alcohol consumption (36.3%), and smoking (32.1%) (Yang et al. 2020a, b).As unhealthy behaviors interact with aging-related vulnerability, understanding how these behaviors cluster and relate to health outcomes is critical for designing effective interventions that promote healthy aging and reduce healthcare demand (Mossey & Shapiro, 2011; Selivanova & Cramm, 2014).

Most existing studies among older Chinese adults have examined single health behaviors (e.g., smoking (Du et al., 2022), diet (Li et al., 2024), drinking (Xia et al., 2024), or health check-ups (Zhao et al., 2022) or specific health outcomes (e.g., functional status (L.-Y. Wang et al. 2023a, b), sleep heath (Liu et al., 2019), diabetes (Bai et al., 2021), or mental health (Feng et al., 2023). However, health behaviors often co-occur and may exert interactive effects on diverse health outcomes (Vermeulen-Smit et al., 2015). Research on behavioral clustering offers a more holistic perspective but remains limited, particularly regarding rural–urban disparities in China (X. Wang et al. 2023a, b; Zeng et al. 2021; Zhang 2021). These disparities, shaped by differences in socioeconomic status, living environments, and healthcare access (Liu et al., 2023), suggest that older adults in rural and urban areas may exhibit distinct health-risk behavior patterns and associated outcomes.

Guided by the Social Ecological Model, which posits that health behaviors are shaped by multiple interacting levels of influence, including individual, interpersonal, community, and societal factors (Mcleroy et al., 1988). This study recognizes that health outcomes reflect both personal choices and broader social and environmental contexts. This framework provides a comprehensive lens to examine how socioeconomic conditions and living environments contribute to behavioral clustering, health disparities, and differences in opportunities for healthy aging among older adults in rural and urban China.

To address these gaps, this study applied latent class analysis (LCA) to identify underlying patterns of health-risk behaviors among rural and urban older adults in China, explored their socioeconomic determinants, and assessed associations with multiple health outcomes, including chronic disease, mental health, functional status, and self-rated health. Using the latest data from the 2018 Chinese Longitudinal Health and Longevity Survey (CLHLS), we hypothesized that distinct subgroups of health-risk behaviors exist across rural and urban populations, reflecting different behavioral profiles and health implications. By integrating behavioral clustering with rural–urban comparison, this study aimed to extend current evidence and offer insights that may inform targeted public health strategies to promote healthy aging.

## Materials and Methods

### Data Source

We used the 2018 dataset from the CLHLS (https://agingcenter.duke.edu/CLHLS), which randomly selects half of the counties and cities from 23 provinces in China for face-to-face interviews with older adults. Since its inception in 1998, CLHLS has collected data across eight waves, with a ninth wave starting in 2021. The dataset includes social, behavioral, environmental, and biomedical variables to support research on aging and health policy. Our study analyzed de-identified data from the 8th wave (2017–2018) with 15,874 participants. After excluding 7,223 ineligible participants (7,171 with incomplete data, 1 educational outlier with an implausible 65 years of education, and 51 under age 65), 8,651 older adults (3,579 rural and 5,072 urban) were included.

### Measures

The selection of seven risky behavior indicators was based on key domains commonly used in health lifestyle research (Du et al., 2022; Li et al., 2024; Xia et al., 2024; Zhang, 2021; Zhao et al., 2022). They were (1) not doing preventive health examinations annually, (2) not engaging in regular exercise at present, (3) smoking at present, (4) using alcohol at present, (5) not eating fresh fruits, (6) not eating fresh vegetables, and (7) not taking nutrient supplements usually. All variables were coded dichotomously, labeling respondents engaging the health risk behaviors as ‘1’ and ‘0’ otherwise, to facilitate the subsequent LCA.

A diverse array of health outcome measures largely aligned with four commonly studied dimensions in prior research is also selected from CLHLS (Zhang, 2021). These dimensions include: (1) physical health indicators, measured by sleep hours (0 = below the recommended threshold of 7 h, 1 = 7 h or more), Body Mass Index (BMI) levels (0 = underweight, 1 = healthy weight, 2 = overweight, 3 = obesity), the number of chronic diseases within the past two years (0 = none, 1 = 1–2 times, 2 = above 2 times), and hearing difficulty (0 = no vs. 1 = yes); (2) perceived health, measured by self-rated health status as an ordinal variable (1 = very bad, 2 = bad, 3 = so-so, 4 = good, 5 = very good), treated as continuous for model simplicity and interpretability (Xu & Shelley, 2023); (3) functional health, assessed by Activity of Daily Living (ADL) disability, defined as difficulty with any of the following: bathing, dressing, eating, indoor transferring, toileting, and continence (0 = no ADL vs. 1 = had ADL); and (4) mental health, including depression and anxiety. Depression was measured using the Center for Epidemiologic Studies Depression Scale, 10-item version (CES-D-10), with a total score ranging from 0 to 30, where higher scores indicate a more depressed mood and scores of 10 and above indicate depression coded 1. Anxiety was measured using the Generalized Anxiety Disorder 7-item (GAD-7) scale, with scores ranging from 0 (not at all) to 3 (nearly every day), and a total score above 8 indicating anxiety coded 1.

The control variables included participants’ age (0 = 65–79, 1 = 80–95, 2 = above 95), gender (0 = female vs. 1 = male), years of education(continuous), marital status (0 = not married vs. 1 = married), living arrangement (0 = alone, 1 = with household member(s), 2 = in an institution), community type (0 = rural vs. 1 = urban), and categorization of province (0 = central south, 1 = east, 2 = north, 3 = northeast, 4 = west). Appendix Table 5 provides a more detailed description for these measures.

### Analytical Approach

This study used LCA to group Chinese older adults based on seven health-risk behaviors.

Unlike traditional factor analysis, which identifies continuous latent factors, LCA identifies categories (classes) of individuals that share similar patterns of behaviors (Byrd & Carter Andrews, 2016). This individual-based approach does not require prior specifications of group distributions and is widely used in social behavior research(Lu et al., 2021). LCA calculated the probability that each participant belongs to each class and the probability of each behavior given class membership (Garnett et al., 2014). This allowed us to identify the main health-risk behaviors, describe unhealthy lifestyle patterns among older adults, and classify participants into subgroups based on their responses.

To find the optimal number of classes, we started with a one-class model and gradually increased the number of classes. We compared models using commonly used fit measures, including the Bayesian Information Criterion (BIC), Akaike Information Criterion (AIC), entropy values, Entropy *(pseudo R²)*, and the Lo-Mendell-Rubin likelihood ratio test (LMR LRT)(Xu et al., 2023). In general, lower AIC and BIC values and higher entropy indicate better model fit, with BIC often considered the most reliable (Byrd & Carter Andrews, 2016). We also considered how interpretable and simple the model was when choosing the final solution.

After selecting the best-fitting model, we created a categorical variable for class membership and assigned each participant to a class based on their health-risk behaviors. To examine what sociodemographic factors predicted class membership, we used multinomial logistic regression. We then analyzed how these classes were associated with health outcomes using different models: ordinary least squares (OLS) regression for self-reported health; binary logistic regression for depression, anxiety, sleep duration, ADL disability, and hearing difficulty; and multinomial logistic regression for the number of chronic diseases and BMI levels.

All analyses were conducted in R (version 4.2.1) using the “*poLCA*” package for LCA, and the “*nnet*” package for multinomial logistic regression.

## Results

### Sample Characteristics

Table 1 presents the descriptive characteristics of study participants (*n* = 8,651). For comparison, Appendix Table 6 shows the characteristics of all participants in the 2018 CLHLS (*n* = 15,874), indicating similar distributions in means and percentages between the two samples. Thus, selection bias is not a concern in this study.

**Table 1:** Descriptive characteristics of CLHLS participants by rural and urban residence

Characteristics			Participants		*P*-value^a^
	Code	Level/range	Rural (*n* = 3579)	Urban (*n* = 5072)	
Age group—no. (%)					
	0	65–79	1551(43.34)	2087(41.15)	**0.04**
	1	80–95	1459 (40.77)	2135 (42.09)	0.217
	2	> 95	569 (15.90)	850 (16.76)	0.287
Gender—no. (%)					**0.005**
	0	Female	1972(55.10)	2638 (52.01)	
	1	Male	1607(44.90)	2434 (47.99)	
Years of education—mean (SD)		[0,20]	2.61(3.34)	4.63(4.83)	**< 0.001**
Marital status—no. (%)					0.361
	0	Not married	1876 (52.42)	2608 (51.42)	
	1	Married	1703(47.58)	2464 (48.58)	
Living arrangement—no. (%)					
	0	Alone	640 (17.88)	795 (15.67)	**0.007**
	1	With household member(s)	2897 (80.94)	4051(79.87)	0.216
	2	In an institution	42 (1.17)	226 (4.46)	**< 0.001**
Province—no. (%)					
	0	Central south	1378 (38.50)	1456 (28.71)	**< 0.001**
	1	East	1599 (44.68)	2091(41.23)	**0.001**
	2	North	75(2.10)	496 (9.78)	**< 0.001**
	3	Northeast	97(2.71)	286(5.64)	**<0.001**
	4	West	430(12.01)	743 (14.65)	**< 0.001**
*Health risky behaviors variables for LCA*					
Doing preventive health examination—no. (%)					**0.006**
	1	No	963(26.91)	1503 (29.63)	
	0	Yes	2616(73.09)	3569 (70.37)	
Exercising—no. (%)					**<0.001**
	1	No	2552 (71.30)	2955 (58.26)	
	0	Yes	1027(28.70)	2117 (41.74)	
Smoking—no. (%)					**0.003**
	1	Yes	632 (17.66)	776 (15.30)	
	0	No	2947 (82.34)	4296(84.70)	
Drinking—no. (%)					0.134
	1	Yes	575 (16.07)	755(14.89)	
	0	No	3004(83.93)	4317 (85.11)	
Eating fresh fruits—no. (%)					**< 0.001**
	1	No	958 (26.77)	987(19.46)	
	0	Yes	2621 (73.23)	4085 (80.54)	
Eating vegetables—no. (%)					0.965
	1	No	94 (2.63)	134 (2.64)	
	0	Yes	3485(97.37)	4938(97.36)	
Taking nutrient supplements—no. (%)					**< 0.001**
	1	No	3259 (91.06)	4299 (84.76)	
	0	Yes	320(8.94)	773(15.24)	
*Health outcome*					
Number of chronic diseases—no. (%)					
	0	None	2722 (76.05)	3776 (74.45)	0.089
	1	1–2	734 (20.51)	1074 (21.18)	0.453
	2	Above 2	123 (3.44)	222(4.38)	**0.028**
Depression —no. (%)					0.091
	0	No depression	2797(78.15)	4040(79.65)	
	1	Depression	782(21.85)	1032(20.35)	
Anxiety—no. (%)					0.491
	0	No anxiety	3447 (96.31)	4899(96.59)	
	1	Anxiety	132(3.69)	173(3.41)	
Self-report health—mean (SD)		[1,5]	3.46(0.88)	3.48 (0.90)	0.272
Sleep duration—no. (%)					0.822
	0	Less than 7 h	1304 (36.43)	1860 (36.67)	
	1	7 h or more	2275(63.57)	3212(63.33)	
BMI—no. (%)					
	0	Under weight	561(15.67)	690(13.60)	**0.007**
	1	Healthy weight	2194(61.30)	2955 (58.26)	**0.005**
	2	Overweight	671 (18.75)	1154 (22.75)	**< 0.001**
	3	Obesity	153(4.27)	273(5.38)	**0.019**
ADL limitations —no. (%)					**< 0.001**
	0	No ADL	3064 (85.61)	4096 (80.76)	
	1	Had ADL	515 (14.39)	976 (19.24)	
Hearing difficulty—no. (%)					0.766
	0	No	2396 (66.95)	3380 (66.64)	
	1	Yes	1183 (33.05)	1692 (33.36)	

^*a*^P-values refer to differences in community residual locations between the ‘rural’ and ‘urban’ groups. These differences are derived from t-tests used to evaluate mean differences in continuous data, and Chi-square tests used to evaluate binary variables. *P* values < 0.05 highlighted in bold

Among the 8,651 participants aged 65 and above, the mean age was 82.95 years (SD = 11.20). Of these, 5,072 (58.63%) lived in urban areas. Rural participants were more concentrated in the younger age group (65–79 years: 43.34%), whereas urban participants were more often in the 80–95 age range (42.09%). More than half of both rural (55.1%) and urban (52.01%) participants were female. Urban participants had substantially more years of education (4.63 vs. 2.61; *p* < 0.001).

Regarding the health behaviors, significant rural–urban differences were observed in preventive health examination (*p* = 0.006), exercise (*p* < 0.001), smoking (*p* = 0.003), fruit consumption (*p* < 0.001), and use of nutrient supplements (*p* < 0.001). Cross-tabulation results indicated that urban participants were more likely to exercise (standardized residual = + 6.38), to take nutrient supplements (+ 5.22), and less likely to abstain from eating fresh fruits (− 4.54).

Differences in certain health outcomes such as BMI and ADL difficulties were also significant (both *p* < 0.001). Rural participants were less likely to be overweight (standardized residual = − 3.06), while urban participants were more likely to experience ADL difficulties (+ 3.45). The prevalence of depression and anxiety did not differ significantly between rural and urban participants (all *p* > 0.05).

#### Latent Class Analysis Results

**Fig. 1:**
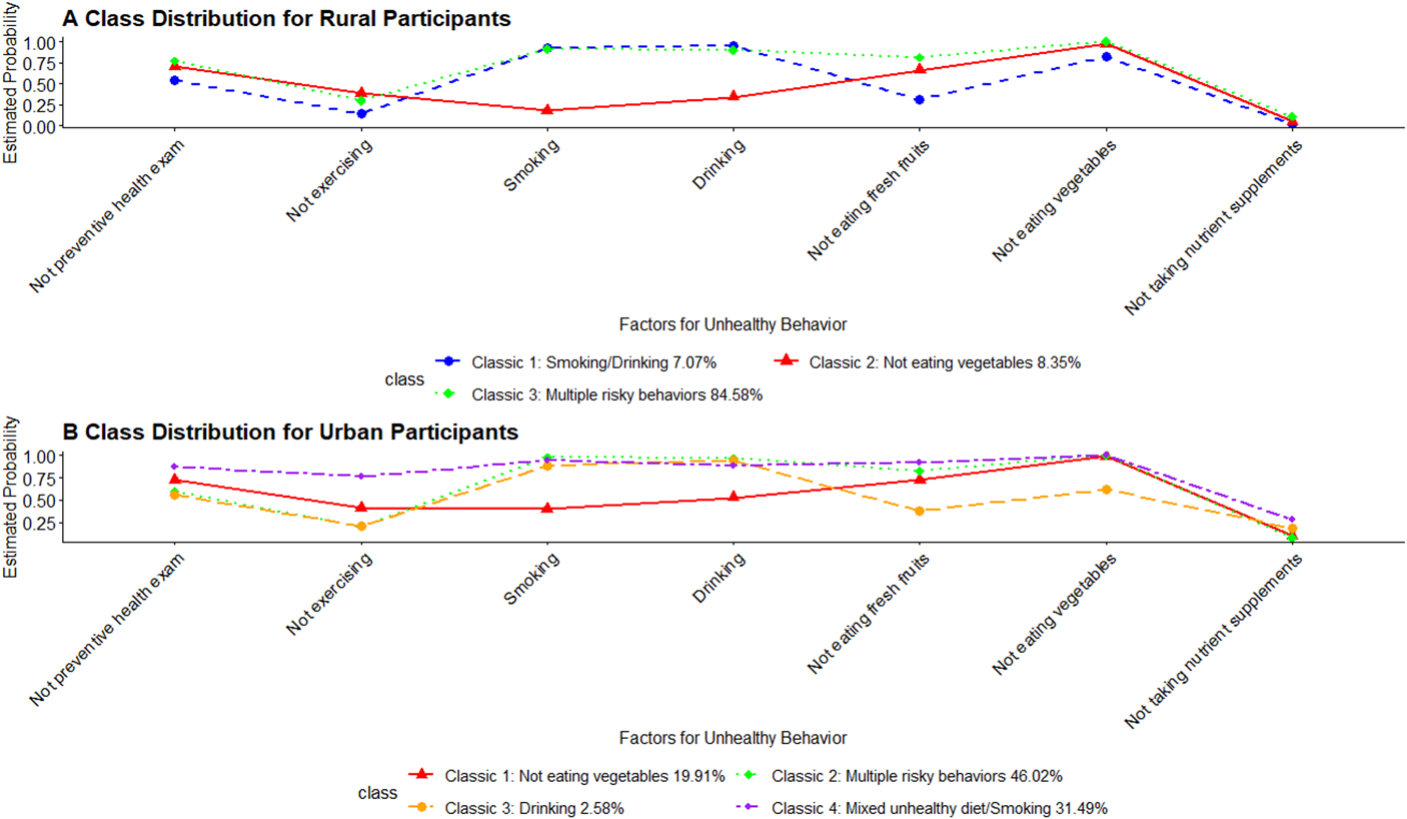
Latent class analysis posterior probability plots for the 7 health-risk behavior indicators

LCA identified three classes for rural participants and four for urban participants based on the lowest BIC values (Table 2). Classes were named according to health-risk behaviors with a posterior probability of ≥ 0.90 (Appendix Table 7). Among rural participants, the three classes were: “*Smoking/Drinking*” (7.07%), “*Not eating vegetables*” (8.35%), and “*Multiple risky behaviors*” (Not eating vegetables/Smoking/Drinking, 84.58%). Among urban participants, the four classes were: “*Not eating vegetables*” (19.91%), “*Multiple risky behaviors*” (Not eating vegetables/Smoking/Drinking, 46.02%), “*Drinking*” (2.58%), and “*Mixed unhealthy diet/Smoking*” (Not eating vegetables/Not eating fresh fruits/Smoking, 31.49%) (Fig. 1).

**Table 2:** Model fitting statistics of latent class analysis

Model	Log-likelihood^a^	Number of parameters^b^	BIC^c^	AIC^d^	Likelihood ratio χ2^e^	Entropy *R*^2f^
Rural participants (*n* = 3579)						
1-Class	−11067.26	7	2191.80	22148.52	577.68	--
2-Class	−10899.59	15	21921.92	21829.18	242.34	0.49
3-Class	−10834.70	23	21857.60	21715.40	112.56	0.45
4-Class	−10819.42	31	21892.52	21700.85	82.01	0.52
Urban participants (*n* = 5072)						
1-Class	−16116.28	7	32292.29	32246.56	872.66	--
2-Class	−15928.12	15	31984.22	31886.24	496.34	0.71
3-Class	−15771.29	23	31738.80	31588.57	182.67	0.39
4-Class	−15722.03	31	31708.53	31506.05	84.15	0.40
5-Class	−15715.84	39	31764.41	31509.68	71.78	0.42

^a^The log-likelihood of the fitted model

^b^The number of parameters estimated by the model

^c^BIC=The Bayesian Information Criterion

^d^AIC=The Akaike Information Criterion

^e^The likelihood ratio chi-squared statistic (G^2)

^f^Entropy of the model, which is a measure of classification certainty

Further descriptive characteristics by new latent classes are presented in Appendix Table 8 (rural) and Appendix Table 9 (urban). Significant differences in sociodemographic and health measures were found between the latent classes for both groups.

#### Significant Predictions of New Class Membership

Table 3 shows that age and gender were significant predictors of latent class membership for both rural and urban participants (*p* < 0.001).

**Table 3:** Multinomial logistic regression models examining the significant predictor of latent classes

		Rural participants (*n = 3579*)		Urban participants (*n = 5072*)		
		Classic 1: Smoking/Drinking vs. Classic 2: Not eating vegetables	Classic 3: Multiple risky behaviors vs. Classic 2: Not eating vegetables	Classic 2: Multiple risky behaviors vs. Classic 1: Not eating vegetables	Classic 3: Drinking vs. Classic 1: Not eating vegetables odds ratios [25%, 75%]	Classic 4: Mixed unhealthy diet/Smoking vs. Classic 1: Not eating vegetables
Characteristics	Value	Odds ratios 95%CI	Odds ratios 95%CI	Odds ratios 95%CI	Odds ratios 95%CI	Odds ratios 95%CI
Age group						
	65–79	Ref	Ref	Ref	Ref	Ref
	80–95	2.85*** [1.81,4.49]	1.74*** [1.30,2.34]	2.07*** [1.71,2.51]	3.88*** [2.24,6.70]	1.52*** [1.25,1.85]
	> 95	7.98*** [3.93,16.19]	3.22*** [1.79,5.78]	2.66*** [2.02,3.51]	5.84*** [3.07,11.10]	0.67* [0.48,0.92]
Gender						
	Female	Ref	Ref	Ref	Ref	Ref
	Male	0.08*** [0.05,0.13]	0.07*** [0.05,0.11]	0.13*** [0.11,0.16]	0.20*** [0.13,0.31]	0.15*** [0.12,0.18]
Years of education	[0,29]	0.89** [0.83,0.96]	1.00 [0.96,1.03]	1.03** [1.01,1.05]	1.02 [0.98,1.08]	1.11*** [1.09,1.14]
Marital status						
	Not married	Ref	Ref	Ref	Ref	Ref
	Married	0.97 [0.61,1.55]	1.21 [0.87,1.69]	0.84 [0.68,1.04]	0.60* [0.36,1.00]	1.03 [0.82,1.28]
Living arrangement						
	Alone	Ref	Ref	Ref	Ref	Ref
	With household member(s)	1.03 [0.62,1.72]	1.01 [0.68,1.50]	1.29* [1.01,1.65]	1.23 [0.74,2.04]	1.10 [0.85,1.42]
	In an institution	1.59 [0.26,9.63]	1.39 [0.31,6.23]	1.38 [0.86,2.21]	0.35 [0.08,1.57]	1.56 [0.95,2.54]
Province						
	Central south	Ref	Ref	Ref	Ref	Ref
	East	0.54** [0.36,0.81]	0.85 [0.64,1.13]	0.97 [0.80,1.18]	0.77 [0.50,1.19]	0.93 [0.75,1.14]
	North	0.77 [0.22,2.65]	0.83 [0.36,1.93]	0.85 [0.62,1.15]	0.66 [0.32,1.34]	1.02 [0.74,1.39]
	Northeast	0.73 [0.24,2.19]	0.73 [0.33,1.62]	0.91 [0.62,1.32]	0.39 [0.13,1.13]	0.99 [0.67,1.47]
	West	0.66 [0.39,1.10]	0.50*** [0.34,0.72]	0.58*** [0.45,0.74]	0.51* [0.28,0.93]	1.08 [0.84,1.39]

Note: Coefficients were relative risk ratio. *p < 0.05; **p < 0.01; ***p < 0.001

Among rural participants, those aged 80–95 and ≥ 95 years were 2.85 and 7.98 times more likely, respectively, to belong to the *Smoking/Drinking* class compared with those aged 65–79. Similarly, the odds of being in the *Multiple risky behaviors* class (Not eating vegetables/Smoking/Drinking) were 1.74 and 3.22 times higher for these age groups.

Among urban participants, gender differences were evident: males had lower odds of belonging to the *Multiple risky behaviors* (OR = 0.13), *Drinking* (OR = 0.20), and *Mixed unhealthy diet/Smoking* (OR = 0.15) classes compared with females, holding other variables constant.

#### Association With Health Outcomes

Table 4 summarizes associations between health risk behavior classes and health outcomes, with detailed results in Appendix Tables 10, 11, 12, 13, 14, 15, 16, 17. *1)Chronic disease*: Compared with the *Not eating vegetables* class, rural participants in the *Smoking/Drinking* class had higher odds of reporting 1–2 chronic diseases (OR = 1.61, 95% CI [1.05, 2.48]), and those in the *Multiple risky behaviors* class had greater odds of reporting ≥ 2 chronic diseases (OR = 2.92, 95% CI [1.16, 7.37]). Similarly, urban participants in other latent classes also showed elevated risks for chronic diseases. *2) Depression and anxiety*: Rural participants in the *Smoking/Drinking* class were more likely to experience depression (OR = 1.73, 95% CI [1.15, 2.63]), while among urban participants, both the *Multiple risky behaviors* (OR = 1.63, 95% CI [1.32, 2.00]) and *Drinking* (OR = 2.08, 95% CI [1.37, 3.15]) classes were associated with higher odds of depression. For anxiety, urban participants in the *Drinking* class had over twice the odds compared to the *Not eating vegetables* class (OR = 2.51, 95% CI [1.16, 5.46]). *3) Weight status*: Among rural participants engaging in *Multiple risk behaviors* and urban participants in the *Mixed unhealthy diet/Smoking* class, the odds of being in the healthy-weight, overweight, and obese categories were higher compared with the *Not eating vegetables* class, suggesting a potential clustering of diet-related and metabolic risks.*4) ADL and hearing difficulties*: Participants in *Multiple risky behaviors* classes also had greater functional and sensory limitations. In rural areas, those in the *Multiple risky behaviors* (OR = 1.93) and *Smoking/Drinking* (OR = 2.81) classes were more likely to have ADL difficulties. In urban areas, the *Multiple risky behaviors* and *Drinking* classes had higher odds of both ADL (ORs = 1.50 and 2.67, respectively) and hearing difficulties (ORs = 1.33 and 1.93, respectively).

**Table 4:** Regression models examining the association of latent classes with various health outcomes

	Rural participants (*n* = 3579)			Urban participants (*n* = 5072)		
Number of chronic diseases						
*Multinomial logistic regression model*	1–2 times vs. None	Above 2 times vs. None		1–2 times vs. None	Above 2 times vs. None	
Class^a^	Odds ratios 95%CI	Odds ratios 95%CI		Odds ratios 95%CI	Odds ratios 95%CI	
Multiple risky behaviors	1.31 [0.95,1.80]	2.92* [1.16,7.37]		1.25* [1.02,1.53]	1.70* [1.12,2.59]	
Smoking/Drinking	1.61* [1.05,2.48]	1.84 [0.54,6.32]				
Drinking				1.52 [0.97,2.38]	3.23** [1.55,6.75]	
Mixed unhealthy diet/Smoking				1.42** [1.15,1.74]	1.68* [1.08,2.60]	
*Depression*^*b*^						
*Logistic regression model*	Depression vs. No depression			Depression vs. No depression		
Class^a^	Odds ratios 95%CI			Odds ratios 95%CI		
Multiple risky behaviors	0.95 [0.69, 1.32]			1.63*** [1.32, 2.00]		
Smoking/Drinking	1.73** [1.15, 2.63]					
Drinking				2.08*** [1.37, 3.15]		
Mixed unhealthy diet/Smoking				0.67*** [0.53, 0.84]		
*Anxiety*^*c*^						
*Logistic regression model*	Anxiety vs. No anxiety			Anxiety vs. No anxiety		
Class^a^	Odds ratios 95%CI			Odds ratios 95%CI		
Multiple risky behaviors	1.02 [0.45, 2.31]			1.26 [0.79, 2.01]		
Smoking/Drinking	2.34 [0.92, 5.95]					
Drinking				2.51* [1.16,5.46]		
Mixed unhealthy diet/Smoking				0.82 [0.48,1.38]		
*Self-reported health*						
*Multiple linear regression model*						
Class^a^	β 95%CI			β 95%CI		
Multiple risky behaviors	−0.04 [−0.15, 0.06]			−0.22*** [−0.29, −0.15]		
Smoking/Drinking	−0.32*** [−0.48, −0.17]					
Drinking				−0.29*** [−0.45, −0.13]		
Mixed unhealthy diet/Smoking				0.08* [0.00,0.15]		
*Sleep duration*						
*Logistic regression models*	7 h or more vs. Less than 7 h			7 h or more vs. Less than 7 h		
Class^a^	odds ratios 95%CI			odds ratios 95%CI		
Multiple risky behaviors	1.26 [0.97,1.62]			0.82* [0.69,0.97]		
Smoking/Drinking	1.00 [0.70,1.44]					
Drinking				1.10 [0.73,1.65]		
Mixed unhealthy diet/Smoking				0.86 [0.72,1.02]		
*BMI*						
*Multinomial logistic regression model*	Healthy weight vs. Underweight	Overweight vs. Underweight	Obesity vs. Underweight	Healthy weight vs. Underweight	Overweight vs. Underweight	Obesity vs. Underweight
Class^a^	Odds ratios 95%CI	Odds ratios 95%CI	Odds ratios 95%CI	Odds ratios 95%CI	Odds ratios 95%CI	Odds ratios 95%CI
Multiple risky behaviors	1.54* [1.07,2.22]	2.27*** [1.44,3.59]	3.29* [1.32,8.16]	1.23 [0.97,1.56]	1.39* [1.04,1.84]	1.32 [0.86,2.02]
Smoking/Drinking	1.06 [0.66,1.68]	1.38 [0.73,2.61]	1.99 [0.62,6.40]			
Drinking				1.00 [0.62,1.62]	1.05 [0.55,1.99]	0.97 [0.34,2.75]
Mixed unhealthy diet/Smoking				1.48** [1.13,1.95]	2.18*** [1.60,2.98]	1.84** [1.18,2.88]
*ADL limitations*						
*Logistic regression models*	Had ADL vs. No ADL			Had ADL vs. No ADL		
Class^a^	Odds ratios 95%CI			Odds ratios 95%CI		
Multiple risky behaviors	1.93 * [1.11,3.36]			1.50*** [1.18,1.91]		
Smoking/Drinking	2.81** [1.49,5.30]					
Drinking				2.67*** [1.69,4.20]		
Mixed unhealthy diet/Smoking				0.80 [0.61,1.05]		
*Hearing difficulty*						
*Logistic regression model*	Yes vs. No			Yes vs. No		
Class^a^	Odds ratios 95%CI			Odds ratios 95%CI		
Multiple risky behaviors	0.96 [0.72,1.29]			1.33** [1.10,1.60]		
Smoking/Drinking	1.22 [0.82,1.81]					
Drinking				1.93** [1.28,2.90]		
Mixed unhealthy diet/Smoking				1.16 [0.95,1.41]		

^a^The reference group for class membership is “*Not eating vegetables*”. Coefficients are relative risk ratio or$\beta$. *p < 0.05; **p < 0.01; ***p < 0.001

^b^Participants with depression score above 10 were categorized into “depression group”, otherwise in “no depression group”

^c^Participants with anxiety score above 8 were categorized into “anxiety group”, otherwise in “not anxiety group”

## Discussion

### General Discussion

In this research, using a large secondary dataset, CLHLS, we observed distinct patterns of health-risk behaviors among older adults in rural and urban areas. Using LCA, we found three latent classes for rural participants and identified four classes for urban participants. Furthermore, class membership was significantly associated with various health outcomes, including higher chances of getting chronic diseases, depression, anxiety, and difficulties in activities of daily living. These findings highlight the need for public health interventions targeting specific high-risk groups in both rural and urban settings.

The results indicate that only age and gender were significant predictors of latent class membership, with older adults and females more likely to engage in high-risk behaviors, potentially due to sociocultural factors. First, males are generally more likely to engage in high-risk behaviors as compared to females(Ashton et al., 2014), largely due to masculinity norms and perceived societal expectations that eventually affect their health behaviors(Mahalik et al., 2007). As a result, men may face a greater risk of acute or chronic health issues(Ashton et al., 2014).

However, the pattern does not hold among older Chinese adults. In contrast, older Chinese women may engage in risky behaviors more frequently than men, influenced by social and psychological factors. Cultural norms and rapid societal changes have left many elderly women in China socially isolated and emotionally neglected, with limited social support(Dong et al., 2009). This isolation, coupled with the challenges of rapid modernization, may lead them to adopt harmful coping mechanisms(H. Yang et al. 2020a, b), such as poor dietary habits, as observed in this study. These behaviors often reflect attempts to manage loneliness and the psychological impacts of aging in a rapidly changing society, especially modernization(H. Yang et al. 2020a, b). Second, the observations regarding older participants rebutted the belief that younger individuals, such as adolescents, are more likely to engage in risk-taking behaviors and make poorer decisions(Balogh et al., 2013). This tendency might be associated with older adults generally experiencing more positive emotions, which makes them more optimistic when assessing risks(Pachur et al., 2017). Additionally, older adults are less discouraged by the potential for losses than younger adults(Pachur et al., 2017).

### Unhealthy Behaviors

We observed that health risk behaviors, particularly drinking and smoking, were associated with worse physical health among rural residents and poorer mental health among urban residents. For example, rural older adults in the “*Smoking/Drinking*” class exhibited 1.61 times more likely to report 1–2 chronic diseases and 2.81 times more likely to have ADL limitation, while those in the combined “*Multiple risky behaviors*” class had nearly triple the odds of reporting more than two chronic diseases and obesity, compared to those who only did not eat vegetables. This pattern is mirrored in urban populations with variations in magnitude, emphasizing the multifaceted effects of lifestyle choices on health conditions(Conry et al., 2011). Moreover, our findings indicated a robust connection between risk behavior classes and mental health outcomes. In rural areas, older adults engaging in smoking and drinking were 73% more likely to suffer from depression, whereas in urban settings, the “*Drinking*” class had more than double the odds of experiencing depression and anxiety. Interestingly, urban residents with a lifestyle of not eating vegetables, not eating fresh fruits, and smoking reported lower rates of depression, suggesting that not all negative behaviors exacerbate health risks (Feng et al., 2023). However, we do not endorse negative behaviors. Cultural beliefs, such as smoking for good luck while playing mahjong, may hinder smoking cessation in China and should be explored further (Lee et al., 2023).

### Policy Implications

The empirical evidence from this present research may lead to preventive measures and interventions. First, public health practitioners should focus on enhancing social support systems for older women, promoting healthier lifestyle choices, and improving access to mental health services. Second, considering that risk behaviors, particularly drinking and smoking, are associated with deteriorated physical health in rural areas and compromised mental health in urban areas, implementing policies that reduce smoking and excessive drinking, alongside promoting nutritional awareness, could significantly enhance health outcomes in the older adult population(Xu et al., 2024).

Last but not least, based on our findings, the primary recommendation should focus on the potential to alleviate urban-rural disparities. China is a country with substantial urban-rural disparities, with rural residents tending to have poorer health outcomes, including smoking and alcohol consumption, cancer diagnosis, healthcare service utilization, and even air-pollution related mortality(Lee et al., 2021; Wang et al., 2022; Xu, 2020; Xu & Shelley, 2023; Zhao et al., 2021). These disparities should be taken into consideration when public health practitioners and policymakers design interventions to promote overall health in Chinese rural areas. Addressing urban-rural health disparities in China remains challenging due to income and education inequalities(Lee et al., 2018), which affect healthcare and preventive care utilization in rural areas. To fully address the potential urban-rural disparities in health, providing a convenient, affordable, and comprehensible approach might be the most effective strategy to support rural residents.

Additionally, our empirical findings should further inform public health interventions at the community level, including culturally adapted smoking cessation programs targeting urban-rural disparities and accessible healthy-eating initiatives. These preventive strategies are consistent with the current public health efforts in China, including the *Healthy China 2030* initiatives. Most importantly, strengthening local engagement at the community level may help reduce behavioral risk factors among Chinese older populations in rural and urban areas.

### Study Limitations

This study is not without limitations. First, as we adopted a cross-sectional design with an observational dataset, we were unable to establish causality for our observations. This was necessary for this research, given that the CLHLS data collection primarily recruited a new group of study participants for the 2018 survey, and the follow-up information may not be available to conduct a longitudinal analysis at this point. Second, the CLHLS data collection process relied on self-reported measurements, which could introduce self-reporting bias, a common limitation in most survey-based research. Third, we included a limited number of health behavior indicators in LCA and examined only a few predictors, which may not capture the full range of relevant factors affecting health outcomes. Fourth, sleep duration was dichotomized using the recommended threshold of 7 h for older adults(Hirshkowitz et al., 2015), to simplify interpretation and reduce skewness; this may have reduced analytical sensitivity, and future studies could treat it as a continuous variable to capture more nuanced associations.

## Conclusion

This study identified distinct latent subgroups of health risk behaviors among urban and rural older adults in China using the LCA method. We uncovered intersecting lifestyle patterns across multiple behaviors, including smoking, drinking, and poor dietary habits. Additionally, we examined significant predictors of class membership and explored the associations between latent classes and various health outcomes. These empirical findings are crucial for designing public health interventions that simultaneously address multiple risk behaviors, especially in areas with limited healthcare access, like rural regions. Public health practitioners and policymakers should continue efforts to reduce urban-rural disparities and address health inequities in the long term.

## Data Availability

The secondary data utilized in this study were extracted from the publicly available data of the Chinese Longitudinal Healthy Longevity Survey (CLHLS).

## Appendix

**Table 5:** Brief introduction of all included variables for 2018 wave for this current study

Variable	Classification	Brief description
*Demographic information*		
Age group	Categorical	Interviewee’s validated age in 2018
	(1) 80–95	
	(2) > 95	
	(3) 65–79 [Ref]	
Gender	Categorical	Gender as reported by the interviewee
	(1) Male	
	(2) Female [Ref]	
Years of education	Numerical	Years of schooling as reported by the interviewee
Marital status	Categorical	Current marital status as reported by the interviewee
	(1) Married	
	(2) Not married [Ref]	
Living arrangement	Categorical	Living arrangement as reported by the interviewee
	(1) With household member(s)	
	(2) In an institution	
	(3) Alone [Ref]	
Province^a^	Categorical	Located province as reported by the interviewee
	(1) East	
	(2) North	
	(3) Northeast	
	(4) West	
	(5) Central south [Ref]	
Community	Categorical	Current residential area as reported by the interviewee
	(1) Urban	
	(2) Rural [Ref]	
*Variable for LCA*		
Preventive health examination	Categorical	Annual and regular physical examinations, as reported by the interviewee
	(1) No	
	(2) Yes [Ref]	
Exercise	Categorical	Current exercise status as reported by the interviewee
	(1) No	
	(2) Yes [Ref]	
Smoke	Categorical	Current smoking experience as reported by the interviewee
	(1) Yes	
	(2) No [Ref]	
Drinker	Categorical	Current alcohol consumption experience as reported by the interviewee
	(1) Yes	
	(2) No [Ref]	
Eating fresh fruits	Categorical	Fresh fruit consumption as reported by the interviewee:
	(1) No	
	(2) Yes [Ref]	
Eating vegetables	Categorical	Vegetable consumption as reported by the interviewee
	(1) No	
	(2) Yes [Ref]	
Taking nutrient supplements	Categorical	Nutrient supplement usage as reported by the interviewee
	(1) No	
	(2) Yes [Ref]	
*Health outcome variable*		
Number of chronic diseases within the past 2 years	Categorical	Number of occurrences of serious illness in the past two years, as reported by the interviewee
	(1) 1–2 times	
	(2) Above 2 times	
	(3) None [Ref]	
Depression	Categorical	Center of Epidemiologic Studies Depression Scale, 10-item version (CES-D-10).
	(1) Depression	
	(2) Not depression [Ref]	
Anxiety	Categorical	Generalized Anxiety Disorder 7-item (GAD-7)
	(1) Anxiety	
	(2) Not anxiety [Ref]	
Self-report health	Numerical	Health status as reported by the interviewee
Sleep duration	Categorical	Daily sleep duration as reported by the interviewee
	(1) 7 h or more	
	(2) Less than 7 h [Ref]	
Body Mass Index (BMI) levels	Categorical	Calculated by dividing the interviewee’s weight in kilograms by their height in meters squared.
	(1) Healthy weight	
	(2) Overweight	
	(3) Obesity	
	(4) Underweight [Ref]	
Activity of Daily Living (ADL)	Categorical	ADL disability was defined as self-reported difficulty with any of the following ADL items as reported by the interviewee: (a) bathing, (b) dressing, (c) eating, (d) indoor transferring, (e) toileting, and (f) continence.
	(1) Had ADL	
	(2) No ADL[Ref]	
Hearing	Categorical	Hearing difficulty as reported by the interviewee
	(1) Yes	
	(2) No [Ref]	

^a^ The participants’ geographical regions were delineated by incorporating the following provinces and megacities: East (Jiangsu, Zhejiang, Anhui, Fujian, Shandong, Jiangxi, Shanghai); North (Tianjin, Shanxi, Hebei, Beijing); West (Shaanxi, Sichuan, Chongqing); Central South (Henan, Hubei, Hunan, Hainan, Guangdong, Guangxi); Northeast (Heilongjiang, Liaoning, Jilin)

**Table 6:** Descriptive statistics comparing the studied dataset (*n* = 8651) with the full sample (*n* = 15,874) in the Chinese longitudinal healthy longevity survey (CLHLS) 2018 wave

Characteristics	Code	Value	Frequency	Percentage	Mean	SD	Missing *n*
Age group— no. (%)							0 (8)
	0	< 65	-- (95)	-- (0.60)			
	1	65–79	3638 (5352)	42.05 (33.72)			
	2	80–95	3594 (6579)	41.54 (41.45)			
	3	> 95	1419 (3840)	16.40 (24.19)			
Gender— no. (%)							0 (0)
	0	Female	4610 (8949)	53.29 (56.38)			
	1	Male	4041 (6925)	46.71 (43.62)			
Years of education— mean (SD)		[0,29] (0,65)			3.80 (3.24)	4.39 (4.30)	0 (2372)
Marital status— no. (%)							0 (267)
	0	Not married	4484 (9196)	51.83 (57.93)			
	1	Married	4167 (6411)	48.17 (40.39)			
Living arrangement— no. (%)							0 (326)
	0	Alone	1435 (2477)	16.59 (15.60)			
	1	With household member(s)	6948 (12497)	80.31 (78.73)			
	2	In an institution	268 (574)	3.10 (3.62)			
Province— no. (%)							0 (0)
	0	Central south	2834 (5760)	32.76 (36.29)			
	1	East	3690 (6408)	42.65 (40.37)			
	2	North	571 (941)	6.60 (5.93)			
	3	Northeast	383 (704)	4.43 (4.43)			
	4	West	1173 (2061)	13.56 (12.98)			
Community— no. (%)							0 (0)
	0	Rural	3579 (7094)	41.37 (44.69)			
	1	Urban	5072 (8780)	58.63 (55.31)			
*Variable for LCA*							
Doing preventive health examination— no. (%)							0 (190)
	1	No	2466 (5117)	28.51 (32.24)			
	2	Yes	6185 (10567)	71.49 (66.57)			
Exercising— no. (%)							0 (349)
	1	No	5507 (10882)	63.66 (68.55)			
	2	Yes	3144 (4643)	36.34 (29.25)			
Smoking— no. (%)							0 (264)
	1	Yes	1408 (2305)	16.28 (14.52)			
	2	No	7243 (13305)	83.72 (83.82)			
Drinking— no. (%)							0 (359)
	1	Yes	1330 (2192)	15.37 (13.81)			
	2	No	7321 (13323)	84.63 (83.93)			
Eating fresh fruits— no. (%)							0 (144)
	1	No	1945 (3900)	22.48 (24.57)			
	2	Yes	6706 (11830)	77.52 (74.52)			
Eating vegetables— no. (%)							0 (133)
	1	No	228 (619)	2.64 (3.90)			
	2	Yes	8423 (15122)	97.36 (95.26)			
Taking nutrient supplements— no. (%)							0 (358)
	1	No	7558 (13771)	87.37 (86.75)			
	2	Yes	1093 (1745)	12.63 (10.99)			
*Health outcome*							
Number of chronic diseases— no. (%)							0 (841)
	0	None	6498 (11035)	75.11 (69.52)			
	1	1–2 times	1808 (3141)	20.90 (19.79)			
	2	Above 2 times	345 (857)	3.99 (5.40)			
Depression— no. (%)							0 (0)
	0	Non-depression	6837 (12685)	79.03 (79.91)			
	1	Depression	1814 (3189)	20.97 (20.09)			
Anxiety— no. (%)							0 (0)
	0	Not anxiety	8346 (15312)	96.47 (96.46)			
	1	Anxiety	305 (562)	3.53 (3.54)			
Self-report health—mean (SD)		[1,5] (1,5)			3.47 (3.43)	0.89 (0.90)	0 (1432)
Sleep duration—no. (%)							0 (891)
	0	Less than 7 h	3164 (5581)	36.57 (35.16)			
	1	7 h or more	5487 (9402)	63.43 (59.23)			
BMI —no. (%)							0 (1302)
	0	Under weight	1251 (2477)	14.46 (15.60)			
	1	Healthy weight	5149 (8573)	59.52 (54.01)			
	2	Overweight	1825 (2796)	21.10 (17.61)			
	3	Obesity	426 (726)	4.92 (4.57)			
ADL limitations—no. (%)							0 (566)
	0	No ADL	7160 (11120)	82.76 (70.05)			
	1	Had ADL	1491 (4188)	17.24 (26.38)			
Hearing difficulty—no. (%)							0 (181)
	0	No	5776 (9263)	66.77 (58.35)			
	1	Yes	2875 (6430)	33.23 (40.51)			

Note: Statistical results for the Full Sample (*n* = 15,874) are provided in parentheses

**Table 7:** Latent class analysis posterior probability plots for rural (*n* = 3579) and urban participants (*n* = 5072)

	Class 1	Class 2	Class 3	Class 4
*Rural Participants (n = 3579)*				
Recoded name	Smoking/Drinking (*n* = 253, 7.07%)	Not eating vegetables (*n* = 299, 8.35%)	Multiple risky behaviors (Not eating vegetables/Smoking/Drinking) (*n* = 3027, 84.58%)	
Doing preventive health exam	0.55	0.70	0.77	
Exercising	0.15	0.38	0.29	
Smoking	**0.92**	0.18	**0.91**	
Drinking	**0.96**	0.34	**0.90**	
Eating fresh fruit	0.31	0.66	0.82	
Eating vegetables	0.82	**0.97**	**1.00**	
Taking nutrient supplements	0.02	0.05	0.11	
*Urban Participants (n = 5072)*				
Recoded name	Not eating vegetables (*n* = 1010, 19.91%)	Multiple risky behaviors (Not eating vegetables/Smoking/Drinking) (*n* = 2334, 46.02%)	Drinking (*n* = 131, 2.58%)	Mixed unhealthy diet/Smoking (Not eating vegetables/Not eating fresh fruits/Smoking) (*n* = 1597, 31.49%)
Doing preventive health exam	0.72	0.59	0.56	0.87
Exercising	0.41	0.20	0.21	0.76
Smoking	0.40	**0.98**	0.88	**0.95**
Drinking	0.52	**0.96**	**0.94**	0.89
Eating fresh fruit	0.72	0.82	0.38	**0.92**
Eating vegetables	**0.99**	**1.00**	0.62	**0.99**
Taking nutrient supplements	0.10	0.08	0.18	0.28

Note: The bold cells (probability is > 0.9) are used to define the classes

**Table 8:** Demographic characteristics of rural participants by new class membership (*n* = 3579)

Characteristics	Code	Value	Classic 1: Smoking/Drinking (*n* = 253)	Classic 2: Not eating vegetables (*n* = 299)	Classic 3: Multiple risky behaviors (*n* = 3027)	*P*-value^a^
Age group—no. (%)						**< 0.001**
	0	65–79	57(22.53)	179(59.87)	1315(43.44)	
	1	80–95	118(46.64)	104(34.78)	1237(40.87)	
	2	> 95	78(30.83)	16(5.35)	475(15.69)	
Gender—no. (%)						**< 0.001**
	0	Female	166(65.61)	28(9.36)	1778(58.74)	
	1	Male	87(34.39)	271(90.64)	1249(41.26)	
Years of education—Mean (SD)		[0,29]	1.29(2.51)	4.24(3.53)	2.56(3.32)	**< 0.001**
Marital status—no. (%)						**0.001**
	0	Not married	178(70.36)	110(36.79)	1588(52.46)	
	1	Married	75(29.64)	189(63.21)	1439(47.54)	
Living arrangement—no. (%)						0.613
	0	Alone	48(18.97)	46(15.38)	546(18.04)	
	1	With household member(s)	201(79.45)	251(83.95)	2445 (80.77)	
	2	In an institution	4(1.58)	2(0.67)	36(1.19)	
Province—no. (%)						**0.001**
	0	Central south	110(43.48)	99(33.11)	1169(38.62)	
	1	East	87(34.39)	132(44.15)	1380(45.59)	
	2	North	5(1.98)	7(2.34)	63(2.08)	
	3	Northeast	7(2.77)	8(2.68)	82(2.71)	
	4	West	44(17.39)	53(17.73)	333(11.00)	
*Health outcome*						
Number of chronic diseases—no. (%)						0.145
	0	None	187(73.91)	239(79.93)	2296(75.85)	
	1	1–2 times	60(23.72)	55(18.39)	619(20.45)	
	2	Above 2 times	6(2.37)	5(1.67)	112(3.70)	
Depression —no. (%)						**< 0.001**
	0	Non-depression	162(64.03)	246(82.27)	2389(78.92)	
	1	Depression	91(35.97)	53(17.73)	638(21.08)	
Anxiety —no. (%)						**0.002**
	0	Not anxiety	234(92.49)	292(97.66)	2921(96.50)	
	1	Anxiety	19(7.51)	7(2.34)	106(3.50)	
Self-report health—Mean (SD)		[1,5]	3.17(0.87)	3.55(0.87)	3.48(0.88)	**< 0.001**
Sleep duration—no. (%)						0.185
	0	Less than 7 h	104(41.11)	115(38.46)	1085(35.84)	
	1	7 h or more	149(58.89)	184(61.54)	1942(64.16)	
BMI—no. (%)						**< 0.001**
	0	Underweight	66(26.09)	46(15.38)	449(14.83)	
	1	Healthy weight	148(58.50)	197(65.89)	1849(61.08)	
	2	Overweight	31(12.25)	50(16.72)	590(19.49)	
	3	Obesity	8(3.16)	6(2.01)	139(4.59)	
ADL limitations—no. (%)						**< 0.001**
	0	No ADL	187(73.91)	283(94.65)	2594(85.70)	
	1	Had ADL	66(26.09)	16(5.35)	433(14.30)	
Hearing difficulty—no. (%)						**< 0.001**
	0	No	134(52.96)	215(71.91)	2047(67.62)	
	1	Yes	119(47.04)	84(28.09)	980(32.38)	

^a^ The p-value was obtained by conducting an ANOVA for numerical variables, and a Chi-squared test for categorical variables. **p* < 0.05; ***p* < 0.01; ****p* < 0.001

**Table 9:** Demographic characteristics of urban participants by new class membership (*n* = 5072)

Characteristics	Code	Value	Classic 1: Not eating vegetables (*n* = 1010)	Classic 2: Multiple risky behaviors (*n* = 2334)	Classic 3: Drinking (*n* = 131)	Classic 4: Mixed unhealthy diet/Smoking (*n* = 1597)	*P*-value^a^
Age group—no. (%)							**< 0.001**
	0	65–79	508(50.30)	737(31.58)	22(16.79)	820(51.35)	
	1	80–95	374(37.03	1028(44.04)	65(49.62)	668(41.83)	
	2	> 95	128(12.67)	569(24.38)	44(33.59	109(6.83)	
Gender—no. (%)							**< 0.001**
	0	Female	200(19.80)	1508(64.61)	75(57.25)	855(53.54)	
	1	Male	810(80.20)	826(35.39)	56(42.75)	742(46.46)	
Years of education—Mean (SD)		[0,29]	4.94(4.52)	3.56(4.53)	3.18(4.52)	6.12(5.05)	**< 0.001**
Marital status—no. (%)							**< 0.001**
	0	Not married	396(39.21)	1437(61.57)	95(72.52)	680(42.58)	
	1	Married	614(60.79)	897(38.43)	36(27.48)	917(57.42)	
Living arrangement—no. (%)							0.084
	0	Alone	153(15.15)	375(16.07)	25(19.08)	242(15.15)	
	1	With household member(s)	826(81.78)	1847(79.13)	104(79.39)	1274(79.77)	
	2	In an institution	31(3.07)	112(4.80)	2(1.53)	81(5.07)	
Province—no. (%)							**< 0.001**
	0	Central south	277(27.43)	713(30.55)	47(35.88)	419(26.24)	
	1	East	412(40.79)	1015(43.49)	53(40.46)	611(38.26)	
	2	North	93(9.21)	207(8.87)	11(8.40)	185(11.58)	
	3	Northeast	51(5.05)	134(5.74)	4(3.05)	97(6.07)	
	4	West	177(17.52)	265(11.35)	16(12.21)	285(17.85)	
*Health outcome*							
Number of chronic diseases—no. (%)							**0.007**
	0	None	791(78.32)	1737(74.42)	89(67.94)	1159(72.57)	
	1	1–2 times	185(18.32)	491(21.04)	31(23.66)	367(22.98)	
	2	Above 2 times	34(3.37)	106(4.54)	11(8.40)	71(4.45)	
Depression —no. (%)							**< 0.001**
	0	Non- depression	846(83.76)	1707(73.14)	90(68.70)	1397(87.48)	
	1	Depression	164(16.24)	627(26.86)	41(31.30)	200(12.52)	
Anxiety —no. (%)							**0.001**
	0	Not anxiety	983(97.33)	2237(95.84)	121(92.37	1558(97.56)	
	1	Anxiety	27(2.67)	97(4.16)	10(7.63)	39(2.44)	
Self-report health—Mean (SD)		[1,5]	3.58(0.89)	3.36(0.90)	3.31(0.95)	3.61(0.88)	**< 0.001**
Sleep durations—no. (%)							**0.0001**
	0	Less than 7 h	315(31.19)	894(38.30)	39(29.77)	612(38.32)	
	1	7 h or more	695(68.81)	1440(61.70)	92(70.23)	985(61.68)	
BMI —no. (%)							**< 0.001**
	0	Under weight	144(14.26)	378(16.20)	30(22.90)	138(8.64)	
	1	Healthy weight	605(59.90)	1382(59.21)	76(58.01)	892(55.85)	
	2	Overweight	213(21.09)	458(19.62)	20(15.27)	463(28.99)	
	3	Obesity	48(4.75)	116(4.97)	5(3.82)	104(6.51)	
ADL limitations—no. (%)							**< 0.001**
	0	No ADL	869(86.04)	1724(73.86)	79(60.31)	1424(89.17)	
	1	Had ADL	141(13.96)	610(26.14)	52(39.69)	173(10.83)	
Hearing difficulty—no. (%)							**< 0.001**
	0	No	716(70.89)	1425(61.05)	61(46.56)	1178(73.76)	
	1	Yes	294 (29.11)	909(38.95)	70(53.44)	419(26.24)	

^a^ The p-value was obtained by conducting an ANOVA for numerical variables, and a Chi-squared test for categorical variables. **p* < 0.05; ***p* < 0.01; ****p* < 0.001

**Table 10:** Multinomial logistic regression models predicting health-related quality of life outcome (number of times suffering from chronic diseases) based on class membership of health-risk behavior indicators

		Rural participants (*n* = 3579)		Urban participants (*n* = 5072)	
		1–2 times vs. None	Above 2 times vs. None	1–2 times vs. None	Above 2 times vs. None
Characteristics	Value	Odds ratios 95%CI	Odds ratios 95%CI	Odds ratios 95%CI	Odds ratios 95%CI
Rural participants					
	Classic 1: Smoking/Drinking	1.61* [1.05,2.48]	1.84 [0.54,6.32]		
	Classic 3: Multiple risky behaviors	1.31 [0.95,1.80]	2.92* [1.16,7.37]		
	Classic 2: Not eating vegetables (Ref)				
Urban participants					
	Classic 2: Multiple risky behaviors			1.25* [1.02,1.53]	1.70* [1.12,2.59]
	Classic 3: Drinking			1.52 [0.97,2.38]	3.23** [1.55,6.75]
	Classic 4: Mixed unhealthy diet/Smoking			1.42** [1.15,1.74]	1.68* [1.08,2.60]
	Classic 1: Not eating vegetables (Ref)				
Age group					
	65–79(Ref)				
	80–95	1.07 [0.88,1.31]	1.01 [0.65,1.58]	1.28** [1.08,1.51]	1.37 [0.97,1.92]
	> 95	0.71* [0.52,0.95]	1.02 [0.54,1.94]	1.13 [0.89,1.43]	1.14 [0.72,1.82]
Gender					
	Female (Ref)				
	Male	1.24* [1.03,1.50]	1.29 [0.85,1.94]	1.17 [1.00,1.37]	1.74*** [1.27,2.37]
Years of education	[0,29]	0.98 [0.95,1.01]	1.00 [0.94,1.06]	0.99 [0.98,1.01]	0.99 [0.96,1.02]
Marital status					
	Not married (Ref)				
	Married	0.99 [0.80,1.22]	1.25 [0.78,2.02]	0.95 [0.80,1.14]	0.72 [0.50,1.02]
Living arrangement					
	Alone (Ref)				
	With household member(s)	1.38* [1.08,1.77]	0.81 [0.49, 1.34]	1.29* [1.04,1.59]	1.15 [0.77,1.72]
	In an institution	1.96 [0.96,4.00]	0.00*** [0.00,0.00]	1.85*** [1.30,2.63]	2.31** [1.24,4.29]
Province					
	Central south (Ref)				
	East	0.99 [0.83,1.19]	0.74 [0.49,1.12]	1.06 [0.90,1.26]	1.22 [0.85,1.74]
	North	1.01 [0.57,1.80]	0.81 [0.19,3.44]	0.96 [0.74,1.24]	0.83 [0.45,1.51]
	Northeast	1.26 [0.77,2.04]	0.31 [0.04,2.30]	1.27 [0.94,1.73]	1.07 [0.53,2.17]
	West	1.00 [0.76,1.31]	2.07** [1.27,3.36]	1.40** [1.13,1.73]	2.72*** [1.84,4.03]

Note: Coefficients were relative risk ratio. Ref=reference group. **p* < 0.05; ***p* < 0.01; ****p* < 0.001

**Table 11:** Binary logistic regression models predicting health-related quality of life outcome (depression) based on class membership of risky healthy behavior

		Rural participants (*n* = 3579)	Urban participants (*n* = 5072)
		Depression vs. Non-depression	Depression vs. Non-depression
Characteristics	Value	Odds ratios 95%CI	Odds ratios 95%CI
Rural participants			
	Classic 1: Smoking/Drinking	1.73** [1.15, 2.63]	
	Classic 3: Multiple risky behaviors	0.95 [0.69, 1.32]	
	Classic 2: Not eating vegetables (Ref)		
Urban participants			
	Classic 2: Multiple risky behaviors		1.63*** [1.32, 2.00]
	Classic 3: Drinking		2.08*** [1.37, 3.15]
	Classic 4: Mixed unhealthy diet/Smoking		0.67*** [0.53, 0.84]
	Classic 1: Not eating vegetables (Ref)		
Age group			
	65–79(Ref)		
	80–95	1.14 [0.93, 1.40]	1.04 [0.88, 1.24]
	> 95	1.16 [0.88, 1.53]	0.81 [0.64, 1.03]
Gender			
	Female (Ref)		
	Male	0.83 [0.68, 1.00]	0.75*** [0.64, 0.89]
Years of education	[0,29]	0.94*** [0.91, 0.97]	0.99 [0.97, 1.01]
Marital status			
	Not married (Ref)		
	Married	0.86 [0.70, 1.07]	0.97 [0.80, 1.16]
Living arrangement			
	Alone (Ref)		
	With household member(s)	0.62*** [0.50, 0.77]	0.74** [0.60, 0.90]
	In an institution	1.23 [0.62, 2.44]	1.35 [0.96, 1.90]
Province			
	Central south (Ref)		
	East	0.79** [0.66, 0.94]	1.01 [0.86, 1.19]
	North	0.56 [0.28, 1.09]	0.46*** [0.34, 0.63]
	Northeast	0.82 [0.49, 1.39]	0.44*** [0.30, 0.66]
	West	0.93 [0.71, 1.21]	0.72** [0.57, 0.91]

Note: Participants with depression score above 10 were categorized into “depression group”, otherwise in “non-depression group”. Ref=Reference group

Coefficients were relative risk ratio. **p* < 0.05; ***p* < 0.01; ****p* < 0.001

**Table 12:** Binary logistic regression models predicting health-related quality of life outcome (anxiety) based on class membership of health-risk behavior

		Rural participants (*n* = 3579)	Urban participants (*n* = 5072)
		Anxiety vs. Not anxiety	Anxiety vs. Not anxiety
Characteristics	Value	Odds ratios 95%CI	Odds ratios 95%CI
Rural participants			
	Classic 1: Smoking/Drinking	2.34 [0.92, 5.95]	
	Classic 3: Multiple risky behaviors	1.02 [0.45, 2.31]	
	Classic 2: Not eating vegetables (Ref)		
Urban participants			
	Classic 2: Multiple risky behaviors		1.26 [0.79, 2.01]
	Classic 3: Drinking		2.51* [1.16, 5.46]
	Classic 4: Mixed unhealthy diet/Smoking		0.82 [0.48, 1.38]
	Classic 1: Not eating vegetables (Ref)		
Age group	65–79(Ref)		
	80–95	0.93 [0.61, 1.43]	0.61* [0.42, 0.89]
	> 95	0.52 [0.27, 1.02]	0.61 [0.36, 1.01]
Gender			
	Female (Ref)		
	Male	0.46*** [0.29, 0.72]	0.65* [0.45, 0.94]
Years of education	[0,29]	0.99 [0.92, 1.06]	0.94** [0.90, 0.98]
Marital status			
	Not married (Ref)		
	Married	1.04 [0.66, 1.64]	0.90 [0.60, 1.35]
Living arrangement			
	Alone (Ref)		
	With household member(s)	0.58* [0.37, 0.91]	0.94 [0.61, 1.45]
	In an institution	0.84 [0.19, 3.73]	0.76 [0.31, 1.85]
Province			
	Central south (Ref)		
	East	0.74 [0.50, 1.09]	0.98 [0.69,1.39]
	North	0.30 [0.04, 2.20]	0.59 [0.28, 1.21]
	Northeast	0.46 [0.11, 1.94]	0.40 [0.14, 1.11]
	West	1.14 [0.67, 1.93]	0.85 [0.51, 1.39]

Note: Participants with anxiety score above 8 were categorized into “anxiety group”, otherwise in “not- anxiety group”. Ref=Reference group. Coefficients were relative risk ratio. **p* < 0.05; ***p* < 0.01; ****p* < 0.001

**Table 13:** Multiple linear regression models predicting health-related quality of life outcome (self-reported health) based on class membership of health-risk behavior

		Rural participants (*n* = 3579)	Urban participants (*n* = 5072)
		Self-reported health	Self-reported health
Characteristics	Value	β 95%CI	β 95%CI
Rural participants			
	Classic 1: Smoking/Drinking	-0.32*** [-0.48, -0.17]	
	Classic 3: Multiple risky behaviors	-0.04 [-0.15, 0.06]	
	Classic 2: Not eating vegetables (Ref)		
Urban participants			
	Classic 2: Multiple risky behaviors		-0.22*** [-0.29, -0.15]
	Classic 3: Drinking		-0.29*** [-0.45, -0.13]
	Classic 4: Mixed unhealthy diet/Smoking		0.08* [0.00,0.15]
	Classic 1: Not eating vegetables (Ref)		
Age group			
	65–79(Ref)		
	80–95	-0.01 [-0.08,0.06]	-0.001 [-0.06,0.06]
	> 95	0.07 [-0.03,0.17]	0.13** [0.05,0.21]
Gender			
	Female (Ref)		
	Male	0.02 [-0.05, 0.09]	0.10*** [0.04,0.15]
Years of education	[0,29]	0.02*** [0.01,0.03]	-0.01** [-0.02,0.00]
Marital status			
	Not married (Ref)		
	Married	-0.06 [-0.13,0.02]	-0.07* [-0.13,0.00]
Living arrangement			
	Alone (Ref)		
	With household member(s)	0.08 [0.00,0.16]	0.04 [-0.03,0.11]
	In an institution	-0.02 [-0.29,0.26]	-0.10 [-0.23,0.03]
Province			
	Central south (Ref)		
	East	0.19*** [0.13,0.25]	0.06 [0.00,0.12]
	North	0.63*** [0.43,0.84]	0.31*** [0.22,0.40]
	Northeast	0.20* [0.03,0.38]	0.36*** [0.25,0.47]
	West	0.03 [-0.07,0.12]	-0.06 [-0.14,0.02]

Note: Coefficients here were not relative risk ratio. They were derived from linear regression models and represent the impact of a predictor variable on the self-reported health outcome variable. Ref=Reference group; **p* < 0.05; ***p* < 0.01; ****p* < 0.001

**Table 14:** Binary logistic regression models predicting health-related quality of life outcome (sleep duration) based on class membership of health-risk behavior

		Rural participants (*n* = 3579)	Urban participants (*n =* 5072)
		7 h or more vs. Less than 7 h	7 h or more vs. Less than 7 h
*Characteristics*	Value	Odds ratios 95%CI	Odds ratios 95%CI
Rural participants			
	Classic 1: Smoking/Drinking	1.00 [0.70, 1.44]	
	Classic 3: Multiple risky behaviors	1.26 [0.97, 1.62]	
	Classic 2: Not eating vegetables (Ref)		
Urban participants			
	Classic 2: Multiple risky behaviors		0.82* [0.69, 0.97]
	Classic 3: Drinking		1.10 [0.73, 1.65]
	Classic 4: Mixed unhealthy diet/Smoking		0.86 [0.72, 1.02]
	Classic 1: Not eating vegetables (Ref)		
Age group			
	65–79(Ref)		
	80–95	0.98 [0.83, 1.17]	1.05 [0.91, 1.21]
	> 95	1.45** [1.14, 1.86]	1.40** [1.14, 1.72]
Gender			
	Female (Ref)		
	Male	1.26 ** [1.07, 1.48]	1.51*** [1.32, 1.73]
Years of education	[0,29]	1.03* [1.00, 1.05]	0.99 [0.98, 1.01]
Marital status			
	Not married (Ref)		
	Married	0.97 [0.81, 1.16]	0.87 [0.75, 1.01]
Living arrangement			
	Alone (Ref)		
	With household member(s)	1.28* [1.05, 1.56]	1.31 ** [1.10, 1.55]
	In an institution	0.78 [0.41, 1.49]	0.87 [0.64, 1.18]
Province			
	Central south (Ref)		
	East	0.91 [0.79, 1.06]	0.86* [0.74, 0.99]
	North	1.69 [0.97, 2.92]	1.03 [0.83, 1.28]
	Northeast	0.69 [0.45, 1.04]	1.02 [0.78, 1.34]
	West	0.76* [0.61, 0.96]	0.96 [0.80, 1.16]

Note: Coefficients were relative risk ratio. Ref=Reference group; **p* < 0.05; ***p* < 0.01; ****p* < 0.001

**Table 15:** Multinomial logistic regression models predicting health-related quality of life outcome (BMI) based on class membership of health-risk behavior

		Rural participants (*n* = 3579)			Urban participants (*n* = 5072)		
		Healthy weight vs. Underweight	Overweight vs. Underweight	Obesity vs. Underweight	Healthy weight vs. Underweight	Overweight vs. Underweight	Obesity vs. Underweight
Characteristics	Value	Odds ratios 95%CI	Odds ratios 95%CI	Odds ratios 95%CI	Odds ratios 95%CI	Odds ratios 95%CI	Odds ratios 95%CI
Rural participants							
	Classic 1: Smoking/Drinking	1.06 [0.66,1.68]	1.38 [0.73,2.61]	1.99 [0.62,6.40]			
	Classic 3: Multiple risky behaviors	1.54* [1.07,2.22]	2.27*** [1.44,3.59]	3.29* [1.32,8.16]			
	Classic 2: Not eating vegetables (Ref)						
Urban participants							
	Classic 2: Multiple risky behaviors				1.23 [0.97,1.56]	1.39* [1.04,1.84]	1.32 [0.86,2.02]
	Classic 3: Drinking				1.00 [0.62,1.62]	1.05 [0.55,1.99]	0.97 [0.34,2.75]
	Classic 4: Mixed unhealthy diet/Smoking				1.48** [1.13,1.95]	2.18*** [1.60,2.98]	1.84** [1.18,2.88]
	Classic 1: Not eating vegetables (Ref)						
Age group							
	65–79(Ref)						
	80–95	0.45*** [0.35,0.59]	0.25*** [0.18,0.34]	0.26*** [0.16,0.41]	0.52*** [0.41,0.66]	0.27*** [0.21,0.35]	0.32*** [0.22,0.46]
	> 95	0.28*** [0.20,0.39]	0.07*** [0.04,0.11]	0.10*** [0.05,0.21]	0.26*** [0.19,0.34]	0.09*** [0.06,0.13]	0.06*** [0.03,0.11]
Gender							
	Female (Ref)						
	Male	1.43** [1.14,1.80]	1.26 [0.95,1.67]	0.89 [0.57,1.37]	1.17 [0.95,1.43]	1.17 [0.93,1.48]	0.76 [0.54,1.07]
Years of education	[0,29]	1.00 [0.96,1.04]	1.04 [0.99,1.09]	1.02 [0.95,1.09]	1.03* [1.01,1.05]	1.03* [1.00,1.05]	1.02 [0.98,1.05]
Marital status							
	Not married (Ref)						
	Married	1.26 [0.98,1.62]	1.25 [0.92,1.70]	0.94 [0.58,1.53]	1.19 [0.94,1.49]	1.32* [1.01,1.73]	1.40 [0.96,2.06]
Living arrangement							
	Alone (Ref)						
	With household member(s)	0.80 [0.61,1.04]	0.86 [0.61,1.22]	0.67 [0.41,1.09]	1.09 [0.86,1.38]	1.02 [0.77,1.37]	1.23 [0.79,1.92]
	In an institution	1.37 [0.50,3.74]	1.92 [0.59,6.26]	1.49 [0.26,8.47]	1.40 [0.88,2.23]	1.68 [0.98,2.87]	1.04 [0.42,2.54]
Province							
	Central south (Ref)						
	East	1.58*** [1.28,1.94]	2.73*** [2.10,3.55]	3.27*** [2.15,4.97]	1.53*** [1.26,1.87]	2.11*** [1.66,2.68]	1.92*** [1.33,2.78]
	North	1.55 [0.70,3.41]	4.17** [1.76,9.86]	4.34* [1.32,14.35]	1.94*** [1.33,2.81]	4.43*** [2.94, 6.68]	6.33*** [3.73,10.73]
	Northeast	1.55 [0.81,2.97]	2.20* [1.02,4.74]	3.68* [1.34,10.11]	1.72* [1.12,2.63]	2.89*** [1.79,4.67]	2.20* [1.08,4.48]
	West	1.70** [1.24,2.33]	1.86** [1.24,2.81]	1.16 [0.53,2.54]	1.36* [1.04,1.77]	1.62** [1.18,2.23]	1.89** [1.18,3.01]

Note: Coefficients were relative risk ratio. Ref=Reference group; **p* < 0.05; ***p* < 0.01; ****p* < 0.001

**Table 16:** Binary logistic regression models predicting health-related quality of life outcome (ADL) based on class membership of health-risk behavior

		Rural participants (*n* = 3579)	Urban participants (*n* = 5072)
		Had ADL vs. No ADL	Had ADL vs. No ADL
*Characteristics*	Value	Odds ratios 95%CI	Odds ratios 95%CI
Rural participants			
	Classic 1: Smoking/Drinking	2.81** [1.49, 5.30]	
	Classic 3: Multiple risky behaviors	1.93 * [1.11, 3.36]	
	Classic 2: Not eating vegetables (Ref)		
Urban participants			
	Classic 2: Multiple risky behaviors		1.50*** [1.18, 1.91]
	Classic 3: Drinking		2.67*** [1.69, 4.20]
	Classic 4: Mixed unhealthy diet/Smoking		0.80 [0.61, 1.05]
	Classic 1: Not eating vegetables (Ref)		
Age group			
	65–79 (Ref)		
	80–95	4.61*** [3.23, 6.60]	4.24*** [3.28, 5.48]
	> 95	17.32*** [11.64, 25.78]	15.03*** [11.20, 20.17]
Gender			
	Female (Ref)		
	Male	1.00 [0.78, 1.29]	1.00 [0.83, 1.21]
Years of education	[0, 29]	0.97 [0.93, 1.02]	0.99 [0.97, 1.01]
Marital status			
	Not married (Ref)		
	Married	0.60*** [0.45, 0.80]	0.53*** [0.43, 0.66]
Living arrangement			
	Alone (Ref)		
	With household member(s)	2.33*** [1.70, 3.20]	2.84*** [2.19, 3.69]
	In an institution	7.56*** [3.51, 16.27]	5.55*** [3.76, 8.18]
Province			
	Central south (Ref)		
	East	2.59*** [2.01, 3.33]	2.15*** [1.74, 2.67]
	North	4.92*** [2.60, 9.33]	3.63*** [2.71, 4.87]
	Northeast	6.10 *** [3.47, 10.74]	6.12*** [4.35, 8.62]
	West	1.97*** [1.39, 2.79]	2.06*** [1.56, 2.71]

Note: Coefficients were relative risk ratio. Ref=Reference group; **p* < 0.05; ***p* < 0.01; ****p* < 0.001

**Table 17:** Binary logistic regression models predicting health-related quality of life outcome (hearing difficulty) based on class membership of health-risk behavior

		Rural participants (*n* = 3579)	Urban participants (*n* = 5072)
		Yes vs. No	Yes vs. No
Characteristics	Value	Odds ratios 95%CI	Odds ratios 95%CI
Rural participants			
	Classic 1: Smoking/Drinking	1.22 [0.82, 1.81]	
	Classic 3: Multiple risky behaviors	0.96 [0.72, 1.29]	
	Classic 2: Not eating vegetables (Ref)		
Urban participants			
	Classic 2: Multiple risky behaviors		1.33** [1.10, 1.60]
	Classic 3: Drinking		1.93** [1.28, 2.90]
	Classic 4: Mixed unhealthy diet/Smoking		1.16 [0.95, 1.41]
	Classic 1: Not eating vegetables (Ref)		
Age group			
	65–79(Ref)		
	80–95	3.16*** [2.60, 3.85]	3.24*** [2.74, 3.83]
	> 95	7.49*** [5.77, 9.72]	10.10*** [8.08, 12.63]
Gender			
	Female (Ref)		
	Male	1.41*** [1.17, 1.68]	1.79*** [1.54, 2.09]
Years of education	[0,29]	0.96** [0.93, 0.98]	0.95*** [0.94, 0.97]
Marital status			
	Not married (Ref)		
	Married	0.66*** [0.54, 0.80]	0.76** [0.64, 0.90]
Living arrangement			
	Alone (Ref)		
	With household member(s)	1.08 [0.88, 1.33]	1.14 [0.94, 1.37]
	In an institution	1.12 [0.57, 2.21]	1.38 [0.99, 1.92]
Province			
	Central south (Ref)		
	East	1.24* [1.04, 1.47]	1.10 [0.94, 1.29]
	North	1.42 [0.83, 2.41]	1.46** [1.14, 1.86]
	Northeast	2.60*** [1.63, 4.13]	1.96*** [1.46, 2.63]
	West	1.42** [1.10, 1.82]	1.39** [1.13, 1.71]

Note: Coefficients were relative risk ratio. Ref=Reference group; **p* < 0.05; ***p* < 0.01; ****p* < 0.001

## Code Availability

Available on request.
